# miRNA-144 targeting DNAJC3-AS1 reverses the resistance of the breast cancer cell line Michigan Cancer Foundation-7 to doxorubicin

**DOI:** 10.1080/21655979.2021.1999373

**Published:** 2021-12-11

**Authors:** Ruiping Ren, Zuguo Yuan, Zhengyang Xu

**Affiliations:** Chemoradiotherapy Center of Oncology, The Affiliated People’s Hospital of Ningbo University, Zhejiang, Ningbo, 315000, RPChina

**Keywords:** MicroRNA-144, DNAJC3-AS1, MCF-7, breast cancer, autophagy, doxorubicin resistance

## Abstract

This study investigated the role of miRNA-144 (miR-144) targeting of the long noncoding DNAJC3-AS1 in regulating breast cancer chemosensitivity. Real-time quantitative polymerase chain reaction was employed to detect the levels of miR-144 in different drug-resistant cells. MTT assays were used to measure the proliferation of cells in different treatment groups. The apoptosis rate of transfected cells was detected by flow cytometry. Western blotting was used to detect levels of DNAJC3-AS1 protein and of autophagy-related proteins. A double luciferase report experiment was performed to evaluate the targeting effect of miR-144 on DNAJC3-AS1. The level of miR-144 was significantly downregulated in MCF-7 doxorubicin-resistant cells. Upregulated expression of miR-144 increased the doxorubicin sensitivity of drug-resistant cells and the rate of apoptosis. DNAJC3-AS1 was the direct target of miR-144; overexpression of DNAJC3-AS1 significantly rescued the apoptosis induced by miR-144 and reversed the inhibition of autophagy by miR-144. Overexpression of miR-144 can reduce drug resistance in breast cancer cells by inhibiting autophagy or targeting DNAJC3-AS1 for downregulation. miR-144/DNAJC3-AS1 provide a new target for reducing drug resistance in breast cancer.

## 1. Introduction

Breast cancer is caused by the malignant transformation of mammary epithelial cells. Options for the clinical treatment of breast cancer include surgical resection, chemotherapy, and radiotherapy; chemotherapy drugs, including doxorubicin (dox), are commonly used [1,2]. At present, despite the development of highly effective and less toxic chemotherapy drugs, resistance to these drugs can develop, leading to a poor prognosis. Therefore, analyzing the molecular mechanisms of dox resistance may allow it to be circumvented, improving the efficacy of the drug. In many mechanistic studies, treatment-induced autophagy has been revealed as a new mechanism of dox-induced antitumor therapy [3].

Autophagy is an evolutionarily conserved process, characterized by the formation of new cells through cell degradation and circulation [4]. Evidence increasingly shows that autophagy can help tumor cells survive outside interference and develop chemotherapy resistance [5–7]. The addition of autophagy inhibitors can improve the sensitivity of cancer cells to chemotherapy [8,9]. It has also been reported that microRNAs (miRNAs) can regulate the sensitivity of tumor cells to chemotherapy and radiotherapy by modulating autophagy [10,11]. Therefore, there is interest in combining autophagy inhibitors with miRNAs as a novel treatment for chemotherapy-resistant cancers.

MicroRNAs (miRNAs) are a class of noncoding small RNA molecules, which can negatively regulate gene expression by destabilizing targeted mRNAs, preventing their translation or targeting them for degradation [12,13]. miR-144 exerts tumor suppressor effects in various tumors and can affect the properties of different tumor cell lines through multiple mechanisms [14]. miR-144 is significantly downregulated in cervical cancer, which can inhibit the proliferation of cervical cancer cell lines and play a role in cancer suppression [15].

Long noncoding RNAs (lncRNAs) participate in the regulation of drug resistance and play an important role in it [16–18]. Among lncRNAs, DNAJC3-AS1 was first discovered in clinical, cisplatin-resistant osteosarcomas to interact with the host gene *DNAJC3*, regulating the cisplatin sensitivity of the cells and resulting in tumors resistant to cisplatin [19]. However, its role in breast cancer is rare. Therefore, we speculated that inhibition of DNAJC3-AS1 activity could reduce drug resistance in breast cancer cells.

In this study, we examined the role of miR-144 in increasing the sensitivity of the human breast cancer cell line MCF-7 to the chemotherapeutic drug doxorubicin and the relationship between miR-144 and DNAJC3-AS1. We show that miR-144 directly targets DNAJC3-AS1 to regulate the dox sensitivity of drug-resistant breast cancer cells.

## 2. Materials and methods

### 2.1. Cell culture

Based on MCF-7 cells (human breast cancer cell line, ATCC, USA) as the material, a drug-resistant cell line (MCF-7/DOX) was established by selecting cells for growth at increasing concentrations of dox (doxorubicin) (Baiolaibo Technology Co., LTD). All cells were cultured in DMEM medium with 10% fetal bovine serum and 1% pentreptomycin in a 5% CO_2_, 37°C incubator. MCF-7/DOX cells were cultured with 1.0 mg/L dox to maintain drug resistance. The cells used in this experiment were approved by the Ethics Committee of the Affiliated People’s Hospital of Ningbo University.

### 2.2. MTT assay

Cell viability was measured using an MTT kit (Gbcbio Technologies Inc.) following manufacturer’s instructions. The cells were plated into 96-well plates, and 10 μL MTT solution was added to each well. The cells were then incubated at 37°C for 2 h. Absorbance at each time point was measured with a microplate reader at 450 nm.

### 2.3. Dual-luciferase reporter assay

We performed a dual-luciferase reporting experiment to determine whether miR-144 regulates the expression of DNAJC3-AS1. Wild-type (WT) and mutant (MT) 3ʹUTR fragments of the gene encoding human DNAJC3-AS1 were amplified from human genomic DNA by polymerase chain reaction (PCR), and a blank control group (NC) was constructed. These, along with the 3ʹUTR of the firefly fluocerite gene (a control), were each inserted into the PGL3 fluorescence vector to create reporter constructs with each 3ʹUTR appended to luciferase. Lipofectamine 2000 was used to transfect cells. After 24 h, the cells were collected and the fluorescence values in each well were determined according to the double fluorescence report analysis kit specifications.

### 2.4. Apoptosis assay

Annexin V-FITC/PI dual-dye combined flow cytometry was used to detect the rate of apoptosis. Cells were treated with dox for 48 h, and then trypsin was added for digestion, and the cell suspension was collected. Annexin V-FITCs 5 μL (Elabscience, Wuhan) was added to each well, and cells were cultured in the dark for 10 min. Then, 10 μL PI was added, and the cells were incubated for another 10 min. The apoptosis rate of cells in each well was measured by flow cytometry (Beckman Coulter).

### 2.5. Cell transfection

miR-144 mimics, miRNA-NC, si-DNAJC3-AS1, pcDNA-DNAJC3-AS1, and the corresponding control (si-NC) of siRNA were purchased from Shanghai Gemma Company. They were diluted with serum-free DMEM before use. Cells were inoculated into a 12-well plate, and cultured for 24 h at 5% CO_2_ and 37°C. Lipofectamine 2000 was then used according to the manufacturer’s instructions. Lipofectamine 2000 was mixed with the diluted noncoding RNAs, added to the cells, mixed well, and incubated for 6 h at 5% CO_2_ and 37°C. The media in each well was then replaced with fresh DMEM with 10% FBS, and the transfected cells were incubated for 48 h at 5% CO_2_ and 37°C.

### 2.6. Quantitative real-time polymerase chain reaction

Cell samples were collected and total RNA (500 ng) was extracted using Trizol reagent (Invitrogen). A mirVana-miRNA Isolation kit (Ambion, Austin, TX, USA) was used to extract miRNA from intestinal tissues. Using a reverse transcription kit (TaKaRa), cDNA was synthesized using input RNA from plasma and intestinal tissues as templates. A SYBR Green Mastermix kit (Takara, Tianjin, China) was used for qPCR, and each experiment was repeated three times. miRNA expression was measured using TaqMan® MicroRNA Assays (Applied Biosystems Inc., ABI) in triplicate. U6 was selected as a reference gene. The PCR reaction program was as follows: 95°C for 30 s, 95°C for 5 s, and 60°C for 30 s with 40 cycles. PCR primer sequences for this experiment included the following: DNAJC3-AS1, 5ʹ–3ʹ: AGCGATTGTGGAAGACCCTG,3ʹ–5ʹ:ATTTCCCCTGGTAAGCGCAA; miR-144, 5ʹ–3ʹ: TCATGTAGTAGATATGACAT,3ʹ–5ʹ:TGGTGTCGTGGAGTCG.

### 2.7. Western blotting

Cell samples were taken, and total protein was extracted from cell lysates. The protein extracts were separated by SDS-PAGE gel electrophoresis, and then transferred to a nitrocellulose membrane. Finally, the membrane was placed in a blocking solution for a 1-h incubation. Monoclonal antibodies (Santa Cruz, USA) against DNAJC3-AS1 (1: 5,000), Agt5 (1: 5,000), LC3 (1: 1,000) were used as primary antibodies to label the target protein. β-actin (1:5,000, Cell Signaling technology, USA) served as a loading control. Secondary antibody (1:1,000, Sigma, USA) was added and incubated at 25°C for 1 h. The membrane was exposed for 5 min in the developer. The band densities were quantified using an infrared imaging system.

### 2.8. Statistical analyses

GraphPad 20.0 was used for data analysis in this study. All experiments were repeated thrice, and the results were expressed as mean ± standard deviation (x ± sd). The chi-square test was used to analyze the comparison between groups. Analysis of variance was used to analyze the differences among groups. The statistical significance threshold was set at *p* < 0.05.

## 3. Results

### 3.1. Expression of miR-144 and DNAJC3-AS1 in MCF-7 and MCF-7/DOX cells

**Figure 1.**
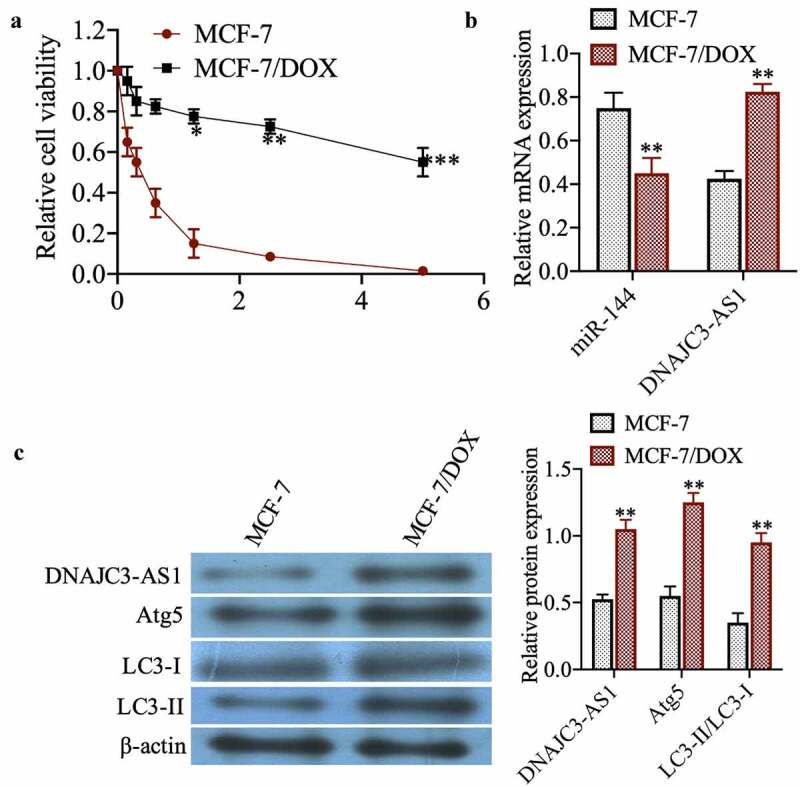
Expression of miR-144 and DNAJC3-AS1 in MCF-7 cells and MCF-7/DOX cells. (a) The relative cell viability was detected by MTT assay. (b) mRNA expression of miR-144 and DNAJC3-AS1 mRNA expression was detected. (c) The expression of DNAJC3-AS1, Atg5, LC3-II and LC3-I protein expression was detected. **P < 0.01vs. MCF-7 group

To investigate the sensitivity of MCF-7 cell lines to dox, drug-sensitive and drug-resistant cells were treated with dox at different concentrations for 48 h. The MTT results show that MCF-7 cells are more sensitive to dox (Figure 1(a)). The expression of miR-144 and DNAJC3-AS1 was then detected by qPCR, showing that miR-144 expression in MCF-7/DOX cells was significantly reduced compared with that in MCF-7 cells, whereas the expression of DNAJC3-AS1 was markedly increased (Figure 1(b)) (*p* < 0.01). Western blotting showed that DNAJC3-AS1, Atg5, and LC3-II/LC3-I were more highly expressed in MCF-7/DOX cells (Figure 1(c)) (*p* < 0.01). In summary, DNAJC3-AS1 and autophagy may be related to drug resistance in MCF-7 cells.

### 3.2. miR-144 overexpression increases dox sensitivity and induces apoptosis of drug-resistant MCF-7 cells

**Figure 2.**
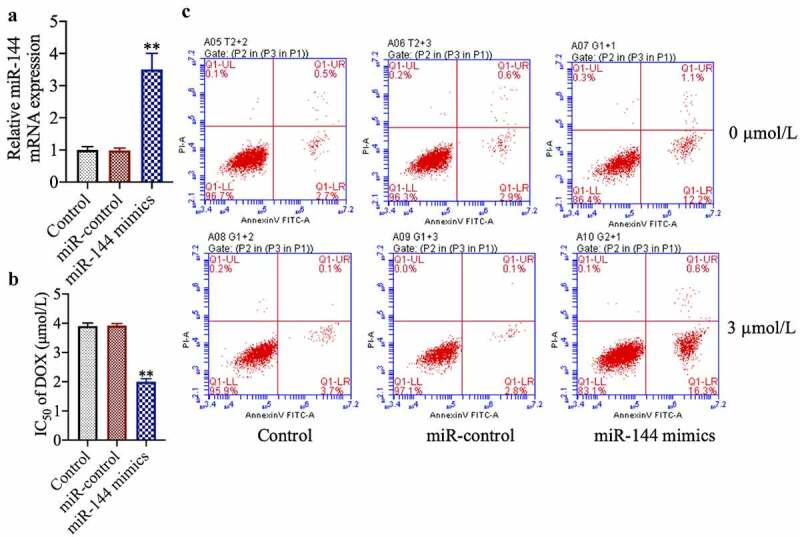
miR-144 overexpression improves the sensitivity and induces apoptosis of drug-resistant MCF-7 cells. (a) The expression of Mir-144 mRNA was detected. (b) Cell viability was detected by MTT. (c) The apoptosis of cells was evaluated by flow cytometry. **P < 0.01 vs. Control group

To study the role of miR-144 in breast cancer chemotherapy resistance, we transfected drug-resistant cell lines with miR-144 mimics. We found that miR-144 expression was increased in the successfully miR-144-mimic-transfected cells (Figure 2(a)) (*p* < 0.01). In addition, we found that transfection of miR-144 mimics also increased the sensitivity of MCF-7/DOX cells to doxorubicin and enhanced the ratio of apoptotic cells (Figures 2(b, c)) (*p* < 0.01).

### 3.3. miR-144 targets and regulates the expression of DNAJC3-AS1, and knocking down the expression of DNAJC3-AS1 increases the dox sensitivity of MCF-7/DOX cells

**Figure 3.**
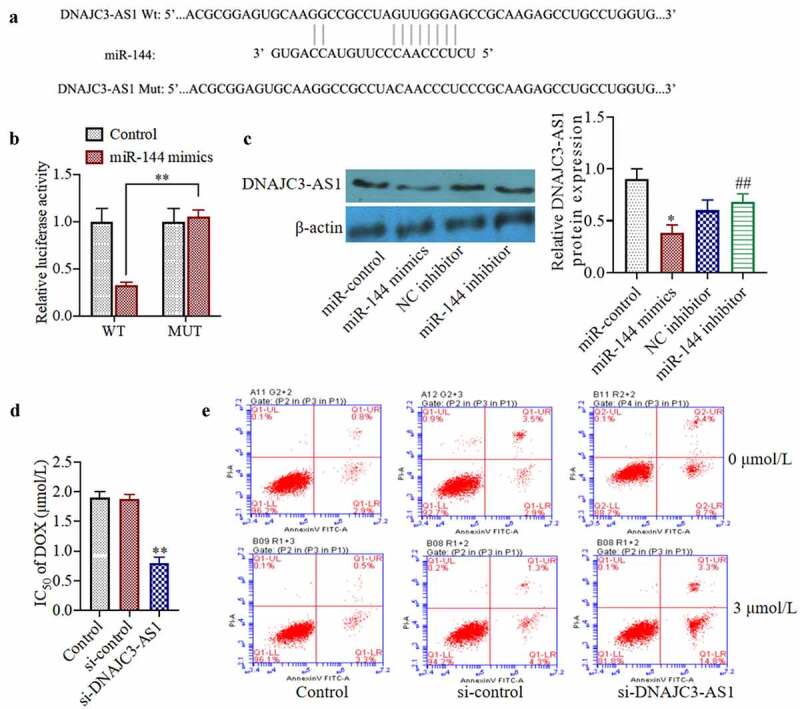
miR-144 targeted regulation of DNAJC3-AS1 expression to detect the sensitivity of drug-resistant MCF-7 cells. (a) Using TargetScan predict the binding sites of miR-144 and DNAJC3-AS1. (b) The targeted relationship between miR-144 and DNAJC3-AS1 was analyzed by Luciferase activity. (c) The DNAJC3-AS1 protein expression was detected. (d) Determination of cell viability. (e) Cell apoptosis was detected. *P < 0.05, **P < 0.01 vs. Control group, ^##^P < 0.01 vs. NC inhibitor

Bioinformatic analysis suggested that miR-144 may target DNAJC3-AS1 (Figure 3(a)). To determine whether miR-144 can directly target DNAJC3-AS1, we conducted a dual-luciferase reporter gene experiment. We found that, compared with a scrambled-sequence control, the miR-144 mimic was able to inhibit wild-type PGL3-WT-DNAJC3-AS1 luciferase activity. When the binding site was mutated, miR-144 mimics had no inhibitory effect on the mutant pGL3-MT-DNAJC3-AS1 luciferase activity (Figure 3(b)). Transfection with miR-144 mimics can also reduce the levels of DNAJC3-AS1 protein in cells (*p* < 0.01) (Figure 3(c)). Moreover, we studied the underlying mechanism of DNAJC3-AS1 by silencing the expression of DNAJC3-AS1 in drug-resistant cells. The results showed that inhibition of DNAJC3-AS1 expression could decrease the resistance of MCF-7/DOX cells to dox and increase the ratio of apoptotic cells (Figure 3(d, e)). The results of this experiment indicate that DNAJC3-AS1 is negatively correlated with the expression of miR-144 in MCF-7 breast cancer cells.

### 3.4. miR-144 increases the chemosensitivity of breast cancer cells by inhibiting the expression of autophagy-related proteins and targeting DNAJC3-AS1

**Figure 4.**
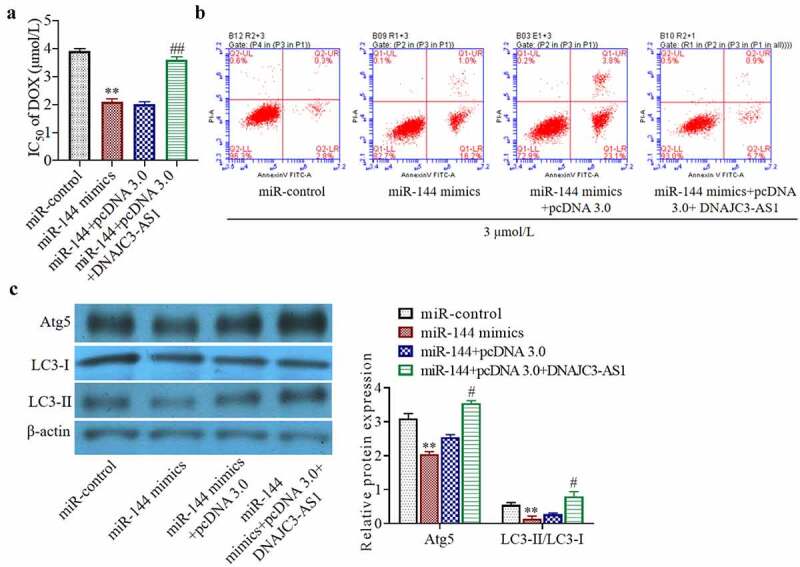
miR-144 increases the chemosensitivity of breast cancer cells by inhibiting the expression of autophagy-related proteins and targeting DNAJC3-AS1. (a) Detection of cell activity. (b) Cell apoptosis was detected. (c) Autophagy related protein expression was detected. **P < 0.01 vs. miR-control group, ^#^P < 0.05, ^##^P < 0.01 vs. miR-144+ pcDNA 3.0 group

To study the mechanism by which miR-144 interacts with DNAJC3-AS1 to regulate drug sensitivity, we chose to overexpress miR-144 or DNAJC3-AS1 in MCF-7/DOX cells. Overexpression of DNAJC3-AS1 significantly dampened the miR-144-mediated increase in dox sensitivity in these cells (Figure 4(a)) (*p* < 0.01). In addition, upregulation of DNAJC3-AS1 partially reversed the pro-apoptotic effect of miR-144 on MCF-7/DOX cells (Figure 4(b)). It can be inferred from the experimental results that miR-144 reduced the resistance of breast cancer cells to doxorubicin by targeting DNAJC3-AS1. Western blot analysis showed that the levels of the autophagy-related proteins Atg5 and LC3-II/LC3-I were significantly decreased by overexpression of miR-144, but this reduction was markedly dampened in the cells simultaneously cotransfected with DNAJC3-AS1 (Figure 4(c)) (*p* < 0.05). In summary, miR-144 reduces the resistance of breast cancer cells to chemotherapeutic drugs by directly targeting DNAJC3-AS1 and inhibiting autophagy.

## 4. Discussion

Doxorubicin (dox) is a chemotherapeutic agent used for various cancers. However, the emergence of adriamycin resistance is also a common clinical event. Studies have shown that drug resistance is related to changes in the miRNA profile of cancer cells [20]. miRNA expression in tumor cells regulates their development and survival [21]. miR-144, in particular, acts as a tumor suppressor in various tumors and can affect the properties of different cancer cell lines by several mechanisms [15]. In this study, we found that miR-144 expression is low in drug-resistant MCF-7/DOX cells, and mRNA and protein expression of DNAJC3-AS1 is high. However, the overexpression of miR-144 reduced the drug resistance of MCF-7/DOX cells and induced apoptosis.

Another study reported that miR-144 can inhibit the expression of *NFE2L2*, thereby reducing the proliferation and metastasis of cervical cancer cells [22]. Gupta et al found that miR-144/199-targeting transcripts of the mesenchymal proteoglycan *Versican* inhibits multiple myeloma by downregulating FAK/STAT3 signal, indicating that miR-144 also affects the FAK/STAT3 signaling pathway, potentially playing the role of tumor suppressor in multiple myeloma as well [23]. Jiang et al found that miR-144 targeting of *APP* transcripts regulates the p-ERK/c-Myc/MMP2 pathway through a phosphorylated extracellular signal to regulate the migration of leukemia cells positive for AML1/ETO fusion protein. This suggests that miR-144 can play a role similar to that of tumor suppressor genes in multiple malignancies [24]. Furthermore, Ma et al found that miR-144 can regulate the tumor-promoting activity of the HOXA10 axis in lung adenocarcinoma; that is, miR-144 also has a tumor suppressor role in lung adenocarcinoma [25]. Finally, Shabani et al found that miR-144, miR-34a, and other miRNAs in the plasma of patients with medullary thyroid cancer are downregulated, possibly promoting the occurrence and progression of thyroid cancer [26]. Through bioinformatics and luciferase reporter experiments, we revealed DNAJC3-AS1 as a target of miR-144 in a breast cancer cell line.

Upregulation of miR-144 can inhibit DNAJC3-AS1 expression. Additionally, we found that silencing DNAJC3-AS1 expression inhibited cellular activity, enhanced dox sensitivity in drug-resistant cells, and promoted their apoptosis. High expression of DNAJC3-AS1 antagonized the pro-dox-sensitivity effect of miR-144, confirming the negative regulatory effect of miR-144 on DNAJC3-AS1. The above results indicate that miR-144 enhances the sensitivity of drug-resistant cell lines to doxorubicin through direct targeting of DNAJC3-AS1.

Autophagy is a physiological process in which cells phagocytose damaged or redundant components and recycle them to maintain a stable intracellular environment [4]. Many studies have found that autophagy plays a dual role in tumor development. Autophagy can not only clear misfolded proteins and inhibit tumor formation but can also aid in the development of tolerance to chemotherapy, thereby promoting the survival of tumor cells [3]. Therefore, we speculate that inhibiting autophagy can overcome drug resistance and improve cancer treatment. Our results indicate that culturing cells with doxorubicin can lead to increased activity of autophagy-associated proteins. Overexpression of DNAJC3-AS1 also promoted an increase in autophagy-related protein levels, whereas an increase of miR-144 effectively reduced this activity. Therefore, overexpression of miR-144 can inhibit autophagy by targeting DNAJC3-AS1 expression, and it significantly increases the ability of adriamycin to induce apoptosis. However, further research is needed to explore in depth the effects of autophagy on drug resistance in cancer cells.

## 5. Conclusion

In summary, miR-144 and DNAJC3-AS1 reciprocally regulate the doxorubicin sensitivity and apoptosis of breast cancer cells and provide new targets for improving the sensitivity of breast cancer cells to chemotherapy in the clinic

In summary, miR-144/DNAJC3-AS1participates in the proliferation and apoptosis of breast cancer cells, and provides a new target for improving the sensitivity of breast cancer to drugs, and has certain application value.
